# Number of chronic diseases and cognitive function among the elderly in China: a moderated mediation model

**DOI:** 10.3389/fpsyg.2025.1491382

**Published:** 2025-03-03

**Authors:** Xiaoling Feng, Jie Peng, Xiaoying Cao, Lichong Lai, Dongmei Huang, Pinyue Tao, Xiao Pan, Qini Pan, Dejing Fan, Shuyu Lu, Caili Li, Yanfei Pan, Pengxin Dong, Haichen Wu, Yidan Chai, Ping Huang, Huiqiao Huang

**Affiliations:** Second Affiliated Hospital of Guangxi Medical University, Nanning, China

**Keywords:** the elderly, chronic diseases, cognitive function, mental health, effect of mediation

## Abstract

**Purpose:**

Despite the wealth of data on the role of chronic disease comorbidity in shaping cognitive dysfunction in older adults, a comprehensive view of this dynamic interplay remains a frontier. This study will reveal the intricate interactions between the number of chronic diseases and cognitive function in the elderly, based on the perspective of cognitive function in patients with multiple chronic diseases.

**Methods:**

Our study was based on the data from the 2023 China Psychological Care for the Elderly Action Survey, and the SPSS 26.0 (IBM Corp., Armonk, NY, United States) software package was used for mediation model analysis. The approach encompassed descriptive analysis of variables, Spearman’s correlation analyses to explore associations between variables, and a moderated mediation analysis.

**Results:**

The study found that the number of chronic diseases (*r* = 0.183, *p* < 0.001) was positively correlated with cognitive function. Anxiety and depression partially mediated the relationship between the number of chronic diseases and cognitive function (*β* = 0.227, 0.235, both *p* < 0.001). Age moderated the association between the number of chronic diseases and depression (*β* = 0.010, *p* < 0.001).

**Conclusion:**

This study provides a comprehensive mediation model that establishes a new association between the number of chronic diseases and cognitive function in older adults. It suggests that we should pay attention to the negative impact of multiple chronic diseases on cognitive function of the elderly and improve their psychological coping ability, so as to ensure the stable development of healthy aging.

## Introduction

With the rapid aging of the global population, elderly health has become a key area of concern. Chronic diseases, such as cardiovascular disease, diabetes, and chronic respiratory disease, are the major factors affecting the physical health and quality of life of older people worldwide (Cong et al., 2024). According to the World Health Organization, the number of people aged 60 years and older is expected to double by 2050, and a large proportion of them will experience multimorbidity, the coexistence of multiple chronic conditions (GBD 2021 Diseases and Injuries Collaborators, 2024). With the change of social economy and lifestyle, the prevalence of chronic diseases in the elderly in China has increased significantly, which has brought major challenges to the medical and health system (Xiang et al., 2024). Although the impact of a single chronic disease on cognitive function has been well documented, the impact of the coexistence of multiple chronic diseases on cognitive health in the elderly has not been fully explored (Ma et al., 2024). Multimorbidity complicates the relationship between chronic diseases and cognitive function, and the impact of multimorbidity on cognitive function may be more serious than that of single chronic disease.

Aging is a natural phase of life that brings about a series of physiological, psychological and social changes. As people age, it is important to take steps to maintain or improve their quality of life for active and healthy aging (Ye et al., 2024). Among the non-pharmacological strategies to improve the quality of life of the elderly, the most recommended is physical exercise, especially strength training, which is an effective intervention (Khodadad Kashi et al., 2023). It can not only improve muscle strength and balance ability, but also help to reduce the frequency of falls, improve the overall physical condition, and reduce the fear of falling, thus helping to improve the quality of life and autonomy of the elderly (Amiri and Sheikholeslami-Vatani, 2023).

In addition to physical health, mental health plays a crucial role in cognitive decline, which raises the question of anxiety and depression. Mental health conditions, particularly anxiety and depression, have been proposed as mediators between chronic diseases and cognitive functioning, but this area remains understudied. Although some studies have touched on the role of anxiety and depression in older people with chronic diseases, the mechanisms by which these mental health conditions affect cognitive function are not fully understood (Meng et al., 2024; Huang et al., 2023). This knowledge gap is significant because it hinders the development of comprehensive intervention strategies.

The present study aimed to address these gaps by examining the number of chronic conditions and how anxiety and depression affect cognitive functioning in older adults. We hypothesized that increasing numbers of chronic diseases would negatively affect cognitive function (Hypothesis H1). This hypothesis is based on the understanding that each additional chronic disease may increase the cumulative burden on the brain’s cognitive reserve, possibly leading to a decline in cognitive performance (Valletta et al., 2023). Second, anxiety and depression could partially mediate this relationship (Hypothesis H2). This is supported by psychological models suggesting that mental health can affect cognitive processes through stress and emotion regulation pathways (Wu et al., 2024). In addition, we will explore whether age moderates the relationship between the number of chronic diseases and depressive status (Hypothesis H3). Age may play a role in this relationship because of age-related biological factors, different coping mechanisms in older versus younger adults, or other factors that influence the expression and impact of depression in the context of chronic disease (Brothers et al., 2021).

By testing these hypotheses, this study will deepen our understanding of the complex interactions between chronic disease, mental health, and cognitive decline and will provide the basis for targeted intervention strategies.

## Methods

### Study participants

This study utilized data from the 2023 National Psychological Care for the Aged Initiative in Guangxi Province, China, aiming to understand the health status of the elderly and related social, behavioral, and biological factors. The study focused on individuals aged 65 and older, which aligns with the conventional definition of ‘elderly’ in China and many other countries. This age range corresponds to an increased prevalence of chronic diseases, marking it as a critical demographic for studying multimorbidity’s impact on cognitive function. A multi-stage stratified sampling method was employed to select 35 districts/counties from 14 prefecture-level cities, ensuring a representative sample across different regions. The stratification was based on a combination of urban vs. rural areas and socioeconomic status, ensuring a representative sample across diverse regions. Within each district/county, the elderly aged 65 years and above were surveyed using a convenience sampling method, which was feasible given the study’s scope and resources. A total of 10,693 interviewees were included, with 323 non-respondents, yielding a response rate of 97.0%.

### Measures

#### Cognitive function

The cognitive function of the elderly was measured by the Cognitive Function Screening tool (AD8) (Galvin et al., 2006). The questionnaire contained 8 items, each of which contained 3 items: change, do not change and do not know. The score for the answer to any question “change” was 1, and the total score was 0–8. If the AD8 total score is ≥2, the possibility of cognitive dysfunction is highly suspected. The Cronbach’s *α* coefficient of the scale in this study was 0.89, which is usually used to screen for cognitive impairment in the elderly.

#### Number of chronic diseases

Information on chronic diseases was obtained by asking participants if they had ever been diagnosed with the following 20 chronic diseases and symptoms, as classified by the International Classification of Diseases, 10th Revision (ICD-10) (Aguado et al., 2020): hypertension, heart disease/coronary heart disease, diabetes mellitus, cerebrovascular disease, chronic bronchitis, cancer, kidney disease, liver disease, gastroenteritis or other gastrointestinal disease, tuberculosis, rheumatoid arthritis, cervical/lumbar disease, reproductive system disease, prostate disease, urinary system disease, glaucoma or cataract, osteoporosis, emotional and mental problems, neurological diseases, and deafness. Each disease was assigned a score of 1, and the total score was summed to quantify the burden of chronic disease per older person. The selection of these diseases was based on their prevalence in elderly populations and their known association with cognitive decline.

#### Level of anxiety

Generalized anxiety Disorder-7 (GAD-7) was used to investigate the subjective feelings of the subjects for 2 weeks, and their anxiety symptoms were scored (Spitzer et al., 2006). The scale had 7 items, each item was scored from 0 to 3, and the total score was from 0 to 21. The score of 0–4 was no anxiety, the score of 5–9 was mild anxiety, the score of 10–14 was moderate anxiety, and the score of 15–21 was severe anxiety. A total score ≥ 5 was considered anxiety symptoms. The Cronbach’s *α* coefficient of the scale in this study was 0.82, which proved the reliability in the elderly population.

#### State of depression

The Patient Health Questionnaire-9 (PHQ-9) was used to measure the depression status of the elderly (Kroenke et al., 2001). The PHQ-9 contains 9 items on a 4-point scale from 0(not at all) to 3(almost every day), with a total score of 0–27. A total score of ≥5 on the PHQ-9 indicates depressive symptoms, and higher scores indicate more severe depressive symptoms. The Cronbach’s *α* coefficient of the scale was 0.88, which was also scientifically applied in the elderly population.

#### Potential confounders

Sociodemographic covariates included sex, place of residence, living arrangements, years of education, and marital status.

#### Statistical analysis

Data analysis was performed using SPSS 26.0 (IBM Corp., Armonk, NY, United States) software package. *T*-test or analysis of variance was used for continuous variables. Correlation analysis was used to determine the potential correlation between the number of chronic diseases, anxiety, depression and cognitive function in the elderly. Subsequently, SPSS macro program PROCESS V3.5 (Igartua and Hayes, 2021) was used to verify the mediating effect of anxiety and depression on the relationship between the number of chronic diseases and cognitive function and the moderating effect of age. All *p*-values are two-tailed, and *p*-values <0.05 were considered statistically significant.

### Study outcomes

#### Common method deviation test

The Harman single factor test method was used to test (Podsakoff et al., 2003), and the results showed that there were 3 factors with eigenvalues greater than 1, and the first factor extracted accounted for 22.53% (<40%) of the variance, indicating that there was no serious common method deviation in this study.

#### Basic information of participants

Among the 10, 370 participants, 44.3% were males and 55.7% were females. 77.5% of the elderly had less than 9 years of education, and 22.5% of the elderly had more than 9 years of education. 51.5% of the elderly lived in urban areas and 48.5% in rural areas. In terms of marital status, most of the respondents had a spouse (70.4%), and those who were separated, divorced or widowed were regarded as having no spouse, accounting for 29.6%. 10.4% of the elderly lived alone, 89.6% of the elderly lived with others (including spouse, children, grandchildren, and other relatives) (Table 1).

**Table 1:** Baseline data and univariate analysis of mental health status (*N* = 10, 370).

Variable	*n*(%)	Depressive state		Anxiety level	
		Score ( $\bar{x}$ ± s)	*T-*value	Score ( $\bar{x}$ ± s)	*T-*value
Gender			−9.528***		−6.787***
Male	4,590 (44.3)	0.98 ± 2.058		0.56 ± 1.708	
Female	5,780 (55.7)	1.41 ± 2.461		0.81 ± 2.007	
Residence			−1.706		1.338
Town	5,337 (51.5)	1.18 ± 2.210		0.73 ± 1.912	
Rural area	5,033 (48.5)	1.26 ± 2.394		0.68 ± 1.856	
Mode of residence			6.906***		4.610***
Live alone	1,083 (10.4)	1.67 ± 2.912		0.95 ± 2.385	
Non solitary	9,287 (89.6)	1.17 ± 2.213		0.67 ± 1.815	
Years of education			41.790***		93.909***
<9 years	8,034 (77.5)	1.22 ± 2.301		0.62 ± 1.347	
≥9 years	2,336 (22.5)	1.07 ± 2.265		0.70 ± 1.885	
Marital Status			−7.614***		−4.158***
Have a spouse	7,302 (70.4)	1.11 ± 2.157		0.65 ± 1.800	
No spouse	3,068 (29.6)	1.48 ± 2.594		0.82 ± 2.068	

***P < 0.001.

### Results of univariate analysis

The general influencing factors of depression and anxiety in 10, 370 elderly people were analyzed, which were gender, residence, living style, years of education, and marital status. The results showed that gender, living style, years of education and marital status were the influencing factors of depression and anxiety in the elderly, and the differences in the scores were statistically significant (*p* < 0.05) (Table 2).

**Table 2:** Means, standard deviations and correlation coefficients of key variables.

Variable	1	2	3	4	5
Cognitive function	–				
Depressive state	0.361***	–			
Anxiety level	0.288**	0.689**	–		
Number of chronic diseases	0.183**	0.242**	0.176**	-	
Age	0.213**	0.086**	0.034**	0.107**	–
Mean value	1.05	1.22	0.70	1.15	73.41
Standard deviation	1.710	2.301	1.885	1.089	6.666

**P < 0.01, ***P < 0.001.

### Descriptive statistics and correlation analysis

Correlation analysis was conducted for the key variables, including the number of chronic diseases, anxiety, depression, cognitive function, and age. The average scores of number of chronic diseases, anxiety and depression were (1.15 ± 1.089), (0.70 ± 1.885) and (1.22 ± 2.301) respectively. The number of chronic diseases (*r* = 0.183, *p* < 0.01), anxiety (*r* = 0.288, *p* < 0.01) and depression (*r* = 0.361, *p* < 0.01) were positively correlated with cognitive function. The number of chronic diseases was positively correlated with anxiety (*r* = 0.176, *p* < 0.01) and depression (*r* = 0.242, *p* < 0.01). Age was positively correlated with anxiety (*r* = 0.034, *p* < 0.01) and depression (*r* = 0.086, *p* < 0.01) (Table 2).

### The mediating effect of anxiety and depression on the number of chronic diseases and cognitive function

**Figure 1:**
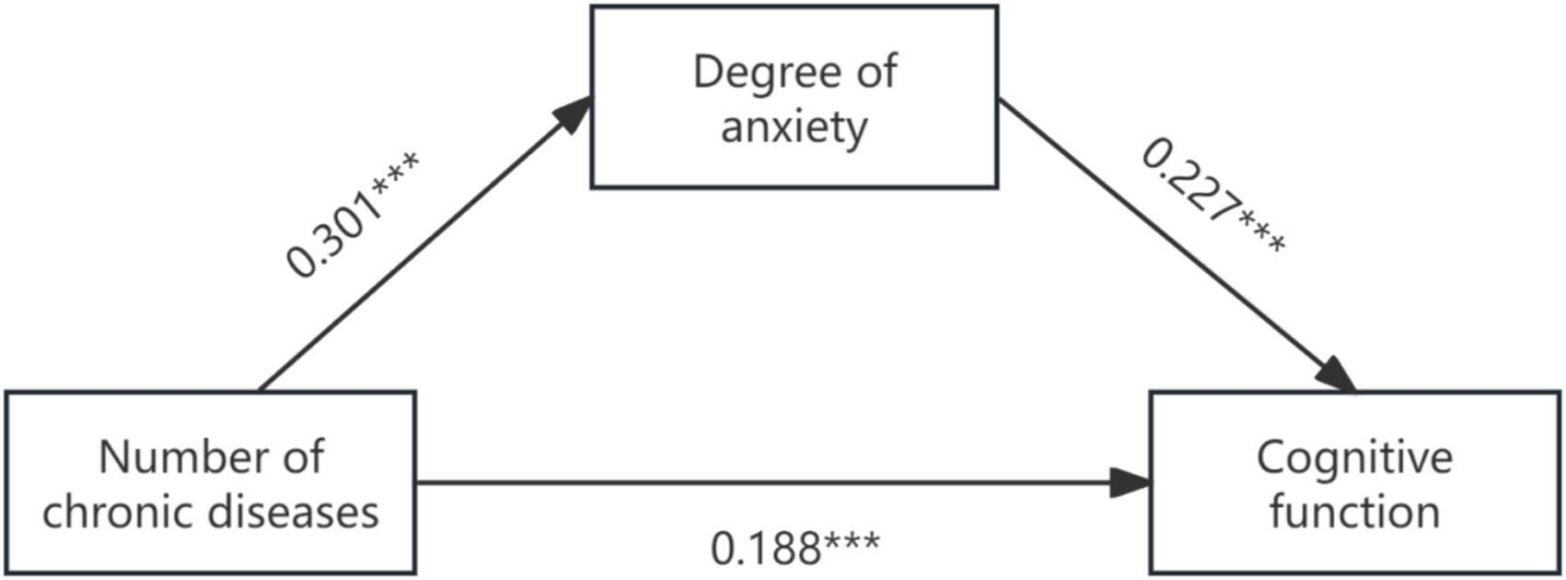
The graph of path coefficients of anxiety level as a mediator of chronic disease number to cognitive function.

First of all, Model 4 of SPSS Process was used to test the mediating effect of anxiety and depression between the number of chronic diseases and cognitive function in the elderly. The results showed that the number of chronic diseases had a significant positive effect on anxiety (*β* = 0.301, *p* < 0.001) and depression (*β* = 0.491, *p* < 0.001) in the elderly. Anxiety (*β* = 0.227, *p* < 0.001) and depression (*β* = 0.235, *p* < 0.001) had a significant positive effect on cognitive function. The number of chronic diseases had a significant positive effect on cognitive function in the mediating model of anxiety and depression (*β* = 0.188, 0.015, both *p* < 0.001). Anxiety and depression partially mediated the relationship between the number of chronic diseases and cognitive function. The results of the specific effect analysis are shown in Table 3, and the mediating effect of anxiety level as a mediating variable is shown in Figure 1.

**Table 3:** The mediating effect of depression on the number of chronic diseases and cognitive function.

Pathway	Effect value (*β*)	BootSE	BootLLCI	BootULCI	Effect size
Anxiety level					
Direct effect	0.1884	0.0145	0.1600	0.2167	73.39%
Indirect effect	0.0683	0.0059	0.0569	0.0803	26.61%
Total effect	0.2567	0.0147	0.2278	0.2856	100%
Depressive state					
Direct effect	0.1423	0.0144	0.1140	0.1705	55.43%
Indirect effect	0.1144	0.0076	0.1001	0.1301	44.57%
Total effect	0.2567	0.0147	0.2278	0.2856	100%

### Moderating effect of age on the association between the number of chronic diseases and depressive status

**Figure 2:**
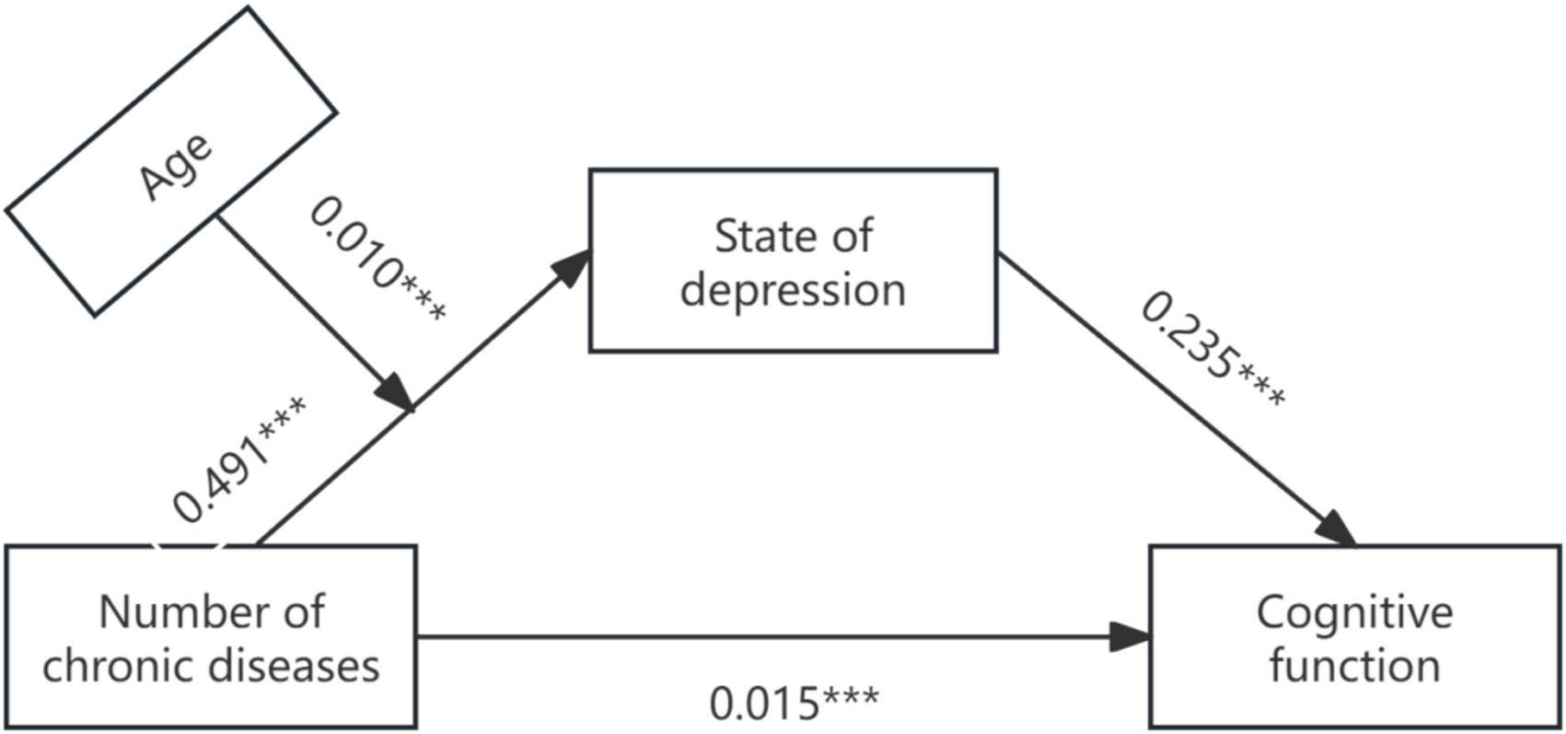
Path coefficient plot of chronic disease and cognitive function mediated by depressive state.

**Figure 3:**
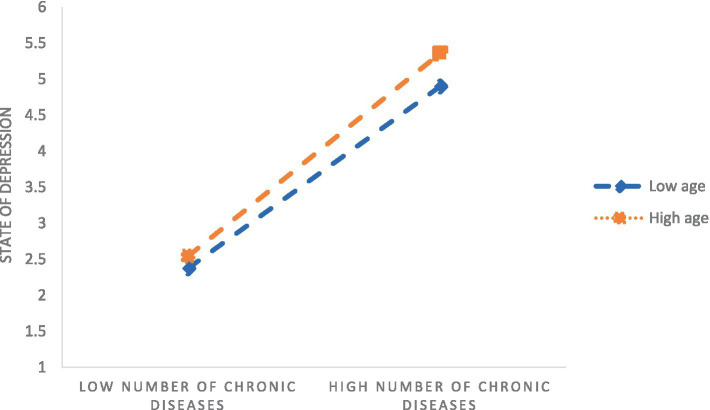
Moderating effect of age on the number of chronic diseases and depressive status.

Secondly, Model 7 in Process (Bootstrap sampling was 5, 000) was used to construct a moderated mediation model with cognitive function as the dependent variable, depression as the mediating variable, and age as the moderating variable. After age was introduced into the model, the product term of the number of chronic diseases and age had a significant positive predictive effect on depression (*β* = 0.010, *p* < 0.001), indicating that age could play a moderating role in the indirect prediction of the number of chronic diseases and depression. The test results of the moderating mediating effect are shown in Table 4, and the moderating mediating effect is shown in Figure 2. Further simple slope analysis was carried out, as shown in Figure 3, in the younger age group, the number of chronic diseases positively predicted depression. In the older age group, the number of chronic diseases still significantly positively predicted depression, but the predictive effect was large, indicating that the predictive effect of the number of chronic diseases on depression gradually increased with age.

**Table 4:** Moderated mediation model testing.

Variable	Depressive state				Cognitive function			
	*β*	SE	*t*	95%CI	*β*	SE	*t*	95%CI
Constant	1.503	0.189	7.956	1.133–1.873	0.220	0.133	1.647	−0.042-0.481
Number of chronic diseases	0.491	0.020	24.373	0.452–0.530	0.164	0.015	11.321	0.136–0.193
Age	0.010	0.004	3.525	0.006–0.019				
Depressive state					0.235	0.007	33.960	0.221–0.248
Number of chronic diseases × Age	0.010	0.003	3.238	0.004–0.016				
*R* ^2^	0.076				0.170			
*F*	121.664				354.539			

## Discussion

In this study, we focused specifically on several potential confounders, including gender, region of residence, lifestyle, years of education, and marital status, as these factors are widely recognized as important factors affecting the number of chronic diseases and cognitive function in older adults. Gender differences play a particular role in the experience of mental health and chronic illness, with studies suggesting that women may be more likely to report depressive symptoms than men (Moreno et al., 2022). This may be attributed to biological factors such as hormonal differences, social factors like gender roles and expectations, or cultural factors that influence help-seeking behavior. In addition, the area of residence is related to the access to health resources and the prevalence of diseases, with urban areas typically having better access to healthcare facilities and preventive services compared to rural areas (Su et al., 2024). Living style, especially living alone versus living with others, is associated with social support and mental health status (Li et al., 2023). Years of education are related to health literacy and disease prevention behaviors, while marital status is related to social support and perceived stress (Stewart et al., 2020; Zhang et al., 2024). By controlling for these confounding factors, we were able to more accurately assess the impact of the number of chronic conditions on cognitive function in older adults and provide more targeted recommendations for public health interventions.

The results of our study indicated that the mean number of chronic diseases among the elderly was 1.15 ± 1.089, a figure that underscores the commonality of multimorbidity in this age group. This finding aligns with prior research, including Hu et al. (2024), which also highlighted the high prevalence of multiple chronic conditions among the elderly. Our data suggest that there is a notable presence of chronic diseases that could be further exacerbated by various stressors, including those related to health crises such as pandemics. However, it is important to note that the direct link between the number of chronic diseases and the pandemic’s impact is beyond the scope of this study and warrants further investigation. The anxiety score of the elderly was (0.70 ± 1.885) points, the depression score was (1.22 ± 2.301) points, and the cognitive function score was (1.05 ± 1.710) points, which were higher than the research results of Peng et al. (2024). This discrepancy could be due to regional differences in mental health awareness and access to mental health services or methodological differences in how anxiety and depression were assessed. Understanding these differences will help the reader interpret our findings more critically.

This study investigated the relationship between the number of chronic diseases and cognitive function in the elderly, and the results showed that the number of chronic diseases significantly positively predicted the cognitive function of the elderly, which verified the hypothesis H1 and was consistent with the research results of Ren et al. (2024) and Lor et al. (2023). In recent years, studies have found that low-grade chronic inflammation, increased frequency of cerebrovascular problems, cumulative effect of disease, and insufficient cerebral oxygen supply may increase the risk of cognitive dysfunction in patients with multiple chronic diseases (Shabir et al., 2020; Burtscher et al., 2021). However, at present, domestic and foreign scholars’ research on the potential mechanisms of cognitive dysfunction in patients with multiple chronic conditions is still in the initial stage of development, and the exact biological pathways of cognitive dysfunction in patients with multiple chronic conditions remain to be elucidated. Researchers need to continue to explore the mechanism of occurrence and development, so as to lay the foundation for clinical multidisciplinary intervention. In addition, the study of Sakakibara et al. (2019) highlighted that the more unhealthy lifestyle factors, the greater the degree of cognitive decline associated with chronic disease comorbidity, and the common pathogenic pathway plays a key role. Specifically, lack of physical activity may increase the vascular and metabolic burden, leading to an increased risk of cognitive dysfunction. Excessive alcohol and smoking can lead to brain damage by promoting vascular damage and inflammatory processes (Freisling et al., 2020), and inflammation may accelerate brain neurodegeneration and vascular pathological changes (Grande et al., 2021), thereby increasing the risk of cognitive dysfunction. Medical staff need to innovate a new self-management model combining self-health monitoring, chronic disease management, and cognitive function prevention according to the characteristics of patients with multiple chronic diseases, and encourage patients with multiple chronic diseases to establish active health thinking, in order to reduce the risk of cognitive dysfunction and improve the quality of life.

This study found that anxiety degree played a partial mediating role between the number of chronic diseases and cognitive function, which confirmed hypothesis H2 and supported the construction model of caring science resilience proposed by Wei et al. (2020). As a key influencing factor of cognitive function, the number of chronic diseases can significantly predict the risk of cognitive impairment in the elderly by affecting the degree of anxiety. This is consistent with previous studies (Teixeira et al., 2015). It can be seen that for the elderly with multiple chronic diseases, it is necessary to pay attention to the change of their anxiety, which is also a favorable measure for the improvement of cognitive function. The results of this study showed that depression partially mediated the relationship between the number of chronic diseases and cognitive function, and age moderated the relationship between the number of chronic diseases and depression. Specifically, the number of chronic diseases in the elderly could significantly predict cognitive function. For older elderly individuals, the predictive effect of the number of chronic diseases on cognitive function was significantly enhanced, which verified hypotheses H2 and H3. In the younger age of the elderly, active prevention and treatment of the occurrence and development of chronic diseases can prevent or delay the risk of cognitive impairment to a certain extent. At the same time, for elderly patients with multiple chronic diseases, more attention should be paid to their mental health status to prevent psychological problems caused by diseases in the elderly (Zhang et al., 2021).

Some limitations of this study need to be addressed. First, only GAD-7, PHQ-9, and AD8 scales are used to measure anxiety, depression, and cognitive function without clinical diagnosis or other cognitive tests, which may not be comprehensive enough. The use of self-reported scales may introduce bias, and clinical diagnoses or other neuropsychological tests could provide more precise measures of cognitive decline. Second, although there is a psychosocial mechanism between chronic disease comorbidity and cognitive function, the mechanism of the vascular mechanism hypothesis has also been proposed (Biessels and Despa, 2018; Pase et al., 2012). However, our study only considered psychological mechanisms as potential mediators, and further research is needed to fully explain the relationship between the number of chronic diseases and cognitive function in older adults. In addition, we recognize that there are some limitations in using data from the Guangxi region to represent the national situation, which may mean that our findings are not fully applicable to all regions of China and need to be further validated with the support of broader national data.

Overall, our findings suggest that mental health should be prioritized in interventions aimed at preventing or managing cognitive decline in older adults with chronic diseases. Public health programs should consider incorporating mental health screenings and support services into chronic disease management plans. Future studies with larger, more diverse samples and longitudinal designs will be essential for further understanding the complex relationship between chronic diseases, mental health, and cognitive function in older adults.

## Data Availability

The original contributions presented in the study are included in the article/supplementary material, further inquiries can be directed to the corresponding author.
